# Bioluminescence as an ecological factor during high Arctic polar night

**DOI:** 10.1038/srep36374

**Published:** 2016-11-02

**Authors:** Heather A. Cronin, Jonathan H. Cohen, Jørgen Berge, Geir Johnsen, Mark A. Moline

**Affiliations:** 1 University of Delaware, School of Marine Science and Policy, Lewes, DE 19958 USA; 2 UiT The Arctic University of Norway, Faculty of Biosciences, Fisheries and Economics, Dept. of Arctic and Marine Biology, N-9037 Tromsø, Norway; 3 University Centre in Svalbard, Pb 156, N-9171 Longyearbyen, Norway; 4 NTNU AMOS–Centre for Autonomous Marine Operations and Systems, Department of Biology, Norwegian University of Science and Technology, N-7491, Trondheim, Norway

## Abstract

Bioluminescence commonly influences pelagic trophic interactions at mesopelagic depths. Here we characterize a vertical gradient in structure of a generally low species diversity bioluminescent community at shallower epipelagic depths during the polar night period in a high Arctic fjord with *in situ* bathyphotometric sampling. Bioluminescence potential of the community increased with depth to a peak at 80 m. Community composition changed over this range, with an ecotone at 20–40 m where a dinoflagellate-dominated community transitioned to dominance by the copepod *Metridia longa*. Coincident at this depth was bioluminescence exceeding atmospheric light in the ambient pelagic photon budget, which we term the *bioluminescence* compensation depth. Collectively, we show a winter bioluminescent community in the high Arctic with vertical structure linked to attenuation of atmospheric light, which has the potential to influence pelagic ecology during the light-limited polar night.

Light and vision play a large role in interactions among organisms in both the epipelagic (0–200 m) and mesopelagic (200–1000 m) realms^1^,^2^. Eye structure and function in these habitats is commonly adapted for photon capture in the underwater light field, with increasing specialization in the mesopelagic^3^. To avoid visual detection, species in epi- and mesopelagic habitats employ cryptic strategies such as transparency^4^ and counter-illumination^5^,^6^, along with diel vertical migration^7^,^8^, to remain hidden from potential predators. In broader physiological terms, the decline in organismal metabolic rate across pelagic depth gradients (and therefore, light gradients) has been related to a decline in visual predation risk and with it a reduction in necessary locomotory capacity^9^,^10^. In all of these cases, the underwater light field impacts pelagic organisms through trophic interactions. While atmospheric light is typically considered to be the dominant source of photons to the underwater light field, chemically-generated light by organisms (bioluminescence) provides an additional source of light to supplement those photons derived from the atmosphere^11^,^12^. This bioluminescence may be solely under the control of the emitting organism, such as in downwelling-directed counter-illumination to help mitigate the shadow of the emitter when viewed from below against downwelling atmospheric light^5^,^6^. Luminescence can also result from physical interactions among individuals, where a swimming organism may mechanically disturb luminescent organisms, causing radially-emitted bioluminescence as either intracellular flashes or extracellular releases. This light can enhance the ability of predators to detect prey (e.g., through the burglar alarm hypothesis^1^,^11^,^12^), or perhaps enhance the ability of prey to detect oncoming predators^13^. Therefore, describing the distribution of bioluminescence throughout the water column and its relative contribution to the pelagic light field is essential to understanding the distribution and behaviors of visual predators and their prey.

At a given depth, the pelagic light field is dependent on the distribution of bioluminescent organisms and the intensity of the bioluminescence they produce upon mechanical stimulation, as well as the intensity of atmosphere-derived light^14^,^15^. Since larger bioluminescent nekton are sparsely distributed in the upper 1000 m of the ocean^16^, the bioluminescent light field is determined by the density and distribution of bioluminescent plankton, which can be patchy or aggregated in thin layers throughout the water column^17^,^18^,^19^,^20^,^21^. Thus, *in situ* bioluminescence measurements are critical to enhancing our understanding of the spatial distribution of bioluminescent assemblages. Approaches have employed a variety of techniques, including *in situ* imaging^22^ and submersible bathyphotometers that pump plankton into an enclosed chamber and measure their emission of light, which is termed *bioluminescence potential*^23^,^24^,^25^,^26^. Such measurements can provide fine-scale resolution of populations of plankton, with sizes ranging from dinoflagellates through meso- and macrozooplankton and micronekton, based on their differential emission intensity and flash kinetics^27^,^28^,^29^ (Fig. 1). This approach is best-suited for use in luminescent communities with relatively low taxonomic diversity, such as thin layers^18^ and polar waters^29^.

In the Arctic, bioluminescence is found throughout the planktonic food web, from dinoflagellate genera *Ceratium* and *Protoperidinium*, through secondary consumers including the euphausiids *Meganyctiphanes* and *Thysanoessa*^29^,^30^,^31^. During spring, summer and autumn, the distribution and community composition of bioluminescent plankton in the Arctic has been investigated using a combination of net sampling and bathyphotometer profiling in fjords, open water, and beneath sea ice. In marginal ice zones, larger mesozooplankton and micronekton contribute the majority of bioluminescence^16^, with other luminescent taxa present and showing depth-dependence, including dinoflagellates at the surface shifting to copepods in deeper waters^23^. However, in coastal fjords, dinoflagellates have been found to dominate the bioluminescence budget with copepods contributing relatively little light^32^.

The Arctic winter during “polar night” presents a dim photic environment that would seem to favor bioluminescent organisms, much like the open ocean mesopelagic habitat^1^,^12^. In contrast to other seasons when atmospheric light is present for some or all of the diel period, the winter light field^33^ would provide far less interference with the production and perception of bioluminescence. The high Arctic during polar night was once understood to be biologically dormant until light and the spring bloom recovered the system with organic carbon through photosynthesis. Currently, increasing evidence is emerging for biological activity across all trophic levels in winter^34^,^35^. Indeed, the only investigations of bioluminescence in the Arctic winter^29^,^31^ have reported extensive luminescence at this time of year in Kongsfjord, Svalbard. However, their sampling was limited to horizontal bathyphotometer transects at 15, 45 and 75 m using an autonomous underwater vehicle^31^, and a 3-day time series buoy-deployment of a bathyphotometer at 20 m depth^29^. Such sampling designs lack the data to fully resolve luminescent community composition over the water column. These studies also lacked ambient light data at this time of year necessary to fully characterize the contribution of bioluminescence to the underwater light field over depth, which is needed to understand what ecological role bioluminescence may be playing in species interactions during the polar night^36^. We do know that zooplankton communities around Svalbard are generally dominated by a few species^37^. Further, they generally decrease both in biomass and species richness during winter, but still with considerable activity^35^, and with some known luminescent taxa such as the copepod *Metridia longa* increasing in abundance as compared to summer^38^.

Given these characteristics, the zooplankton community in winter is an ideal system for studying the community composition and distribution of bioluminescent plankton. With low ambient light (irradiance, E) at these locations (e.g., E_PAR_ < 3.5 ×10^−5 ^μmol m^−2 ^s^−1^ at surface during solar noon in January^33^), bioluminescence may contribute a greater proportion of photons to the pelagic light budget than atmospheric light. To test this, we used vertical bathyphotometer profiles to (1) measure bioluminescence potential in a Svalbard fjord during the Arctic winter, (2) determine luminescent community composition as a function of depth from luminescence flash kinetic signatures, and (3) quantify the contribution of luminescence to the pelagic light budget.

## Results

### Water column physical properties

During profiling in Kongsfjord, mean water temperature ranged between −0.2 and 0.8 °C throughout the water column, while mean salinity ranged between 34.67 and 34.86. The coldest temperatures and lowest salinities were above 30 m (Fig. 2a). Mean seawater density as sigma-theta (σ_θ_) was between 27.85 and 27.93 for all depths below 1 m. Downwelling irradiance (E_d_) in the water column modeled based on the measured late-January atmospheric light field and IOPs (Fig. 2a,b) had a spectral transmission maximum of 455 nm, and integrated downwelling E_PAR_ of 3.5 × 10^−5 ^μmol photons m^−2^ s^−1^ at the surface. By 99 m, maximum transmission had shifted to 495 nm and E_PAR_ decreased to 5.5 × 10^−13 ^μmol photons m^−2^ s^−1^.

### Bioluminescence

**Figure 1:**
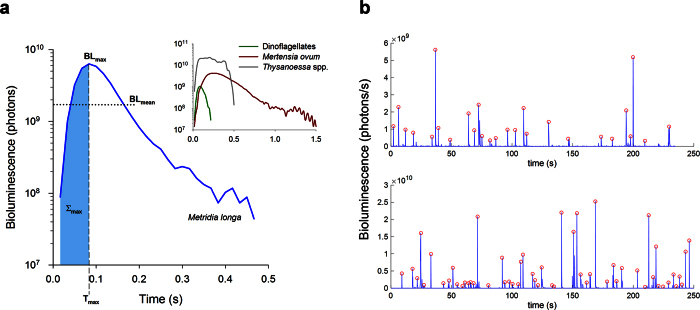
Flash kinetics for bioluminescent emissions of plankton recorded in a bathyphotometer. (**a**) Shown is a representative luminescent emission from the copepod *Metridia longa* (blue line), with parameters we used to create taxonomic signatures for *in situ* identifications of this and other luminescent plankton (see Table 1). Parameters are maximum bioluminescence (BL_max_), average bioluminescence produced during the emission (BL_mean_), time until the emission reached maximum (T_max_), and cumulative sum of bioluminescence produced until the maximum (Σ_max_; blue shading). Inset are representative luminescent emissions from dinoflagellates (green line), ctenophores *Mertensia ovum* (red line), and krill *Thysanoessa* spp. (grey line). (**b**) Examples of underwater bathyphotometer (UBAT) time series, showing individual flashes flagged with red circles at their peaks. These data are from the same noontime cast, with the upper panel showing a 4 min stop at 1 m, and the lower panel a 4 min stop at 80 m. Note the difference in scale, and in turn flash intensity, between these two depths across an ecotone (see Results).

**Figure 2:**
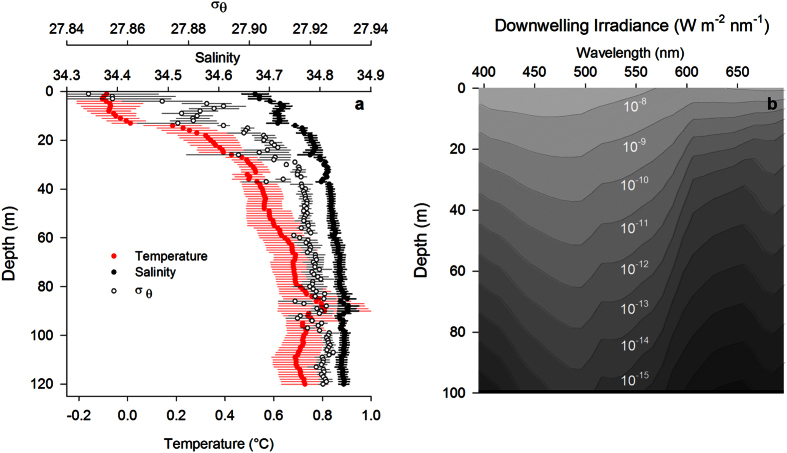
Water column physical characteristics in Kongsfjord. (**a**) Mean profiles (n = 3) of salinity, temperature and σ_θ_ (±SE) taken by CTD (2014) concurrent with measurements of bioluminescence potential. (**b**) Downwelling spectral irradiance modelled in Kongsfjord for midday on 25 January 2015 as described in Methods.

**Figure 3:**
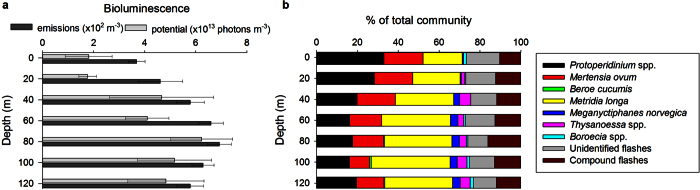
Bioluminescence profiles in Kongsfjord. (**a**) Profiles of mean bioluminescence potential (±SE, n = 4) measured for 4 minutes at 20 m intervals in January 2014. Bioluminescence potential is represented as total number of emissions m^−3^ (dark bars) and in number of photons (x10^13 ^m^−3^) produced (light bars). (**b**) Relative proportion of the bioluminescent community (calculated from the number of individual emissions, which were then identified to species as described in Methods) comprised by each known bioluminescent taxonomic group and by unidentified individuals. Compound flashes which were not processed using the bioluminescence library are also included.

We profiled an underwater bathyphotometer (UBAT) in the water column, with 4 min stops at 20 m depth intervals (e.g., Fig. 1b), to measure bioluminescence potential and determine bioluminescent community composition in Kongsfjord during January 2014. The number of bioluminescent emissions during profile stops increased with increasing depth to a maximum of 693 emissions m^−3^ at 80 m (Fig. 3a). There were significantly fewer emissions at 1 m depth than at 40 m and greater (ANOVA, F_6,27_ = 5.661, *P* = 0.001; Holm-Sidak post-hoc tests, α = 0.05). Mean bioluminescence potential similarly increased with depth to a maximum of 6.2 × 10^13 ^photons m^−3^ at 80 m (Fig. 3a), but the only significant difference among depths was between 1 m and 80 m (ANOVA, F_6,27_ = 3.589, *P* = 0.013; Holm-Sidak post-hoc tests, α = 0.05). The greatest difference in both mean number of emissions and bioluminescence potential between consecutive depth intervals occurred between 20 m and 40 m (Fig. 3a).

Using the UBAT in the laboratory, we tested 17 taxa for emission of bioluminescence. Of these, only seven taxa emitted bioluminescence, each with a distinct flash kinetic signature (Table 1), which we then used to identify taxa responsible for the emissions recorded in our UBAT profiles from Kongsfjord (Table 2). Dinoflagellates (*Protoperidinium* spp.) comprised the greatest proportion of the bioluminescent community at the shallowest depths sampled (1 m and 20 m) and copepods (*Metridia longa*) contributed the greatest proportion at 40 m and deeper (Fig. 3b). Among the bioluminescent community, only *M. longa* varied in abundance among depth intervals, being significantly more abundant at 80 m and 100 m than they were at 1 m or 20 m (ANOVA, F_6,27_ = 7.321, *P* < 0.001). Among the lesser abundant taxa, 15–19% of organisms in the bioluminescent community identified based on flash kinetics was composed of the ctenophore *Mertensia ovum* above 80 m, while it contributed 10–13% below 80 m (Fig. 3b). Krill *Thysanoessa* spp. and *Meganyctiphanes norvegica* were variable in their contribution to the bioluminescent community. Krill are not sampled well by the UBAT^29^,^31^ because of their relatively strong swimming ability and the intake avoidance it confers^39^,^40^,^41^, as well as their light-induced intracellular luminescence used in counter-illumination as opposed to mechanically-stimulated extracellular luminescence found in taxa like *Metridia*. Thus, while neither krill species exceeded 6% of the community at any depth, and both were lowest in their contribution in the upper 20 m (Fig. 3b), these are likely underestimates for krill abundance, but not necessarily for luminescence production by mechanical stimulation. At any depth, compound and unidentified flashes each constituted 16% or less of total emissions (Fig. 3b).

**Figure 4:**
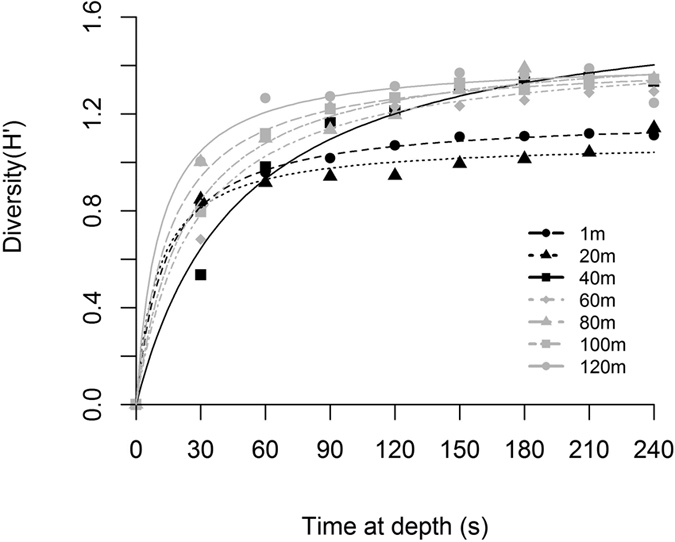
Diversity of the Kongsfjord luminescent community. Mean (n = 4, symbols) Shannon diversity (H′) at every 30s for each depth interval over the 4-minute (89.5 L) sampling period for profiles (see Fig. 3). Overlaid, is a nonlinear regression model for each depth (lines). Grey symbols/lines represent communities at 60 m and below, while dark symbols/lines represent communities at 40 m and above.

With a 4-min pumping period at each depth interval, we found differences among all depths for Shannon diversity (H′) and Pielou’s evenness (J′) indices of the bioluminescent community determined by the flash kinetic signatures (ANOVA for each index, F_6,27_ = 3.363, *P* = 0.018) (Table 3). However, differences among individual depths were not apparent for either index with multiple comparisons testing (Holm-Sidak post-hoc tests, *P* > 0.05 for all depth comparisons within each index). When we calculated diversity at 30 s intervals over the 4-min pumping period, the depth-stratified bioluminescent communities reached maximum diversity at different rates (Fig. 4). In nonlinear regression models, the community at 40 m was both the most diverse (i.e. largest H′_max_) and the slowest to reach H′_max_ (i.e. largest K), while communities at 1 m and 20 m were the least diverse (Table 3).

## Discussion

**Figure 5:**
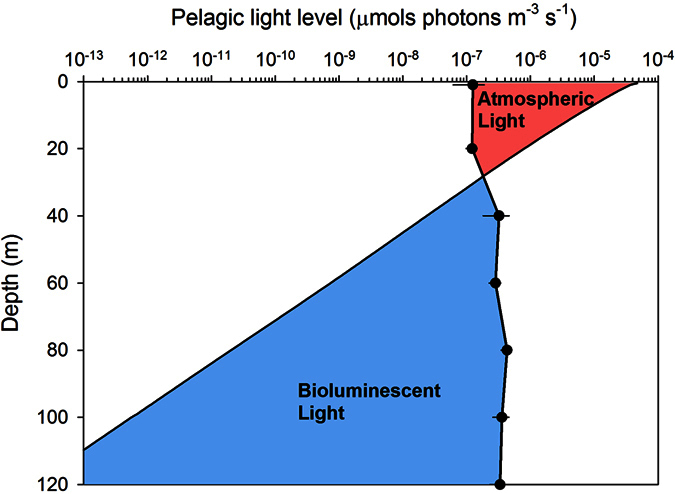
Photon budget for biological and atmospheric light sources in Kongsfjord. The two components of the underwater light field in Kongsfjord are plotted as a function of depth in equivalent units (μmol photons m^−3^ s^−1^). Atmospheric light is scalar irradiance (solid line; 400–700 nm, E_o,PAR_) modeled from diffuse atmospheric irradiance measured at approximately solar noon on 25 January 2015. Bioluminescence is mean bioluminescence potential (±SE, black dots) measured at midday and midnight in January 2014. The colors represent photic zones dominated by atmospheric light (red shading) and bioluminescent light (blue shading).

The underwater light field impacts the distribution and behavior of pelagic organisms in aquatic habitats globally^8^,^33^. In Kongsfjord during polar night, the luminescent community composition transitioned between 20 m and 40 m, and concomitantly the source of photons changed from atmospheric light to bioluminescence (Fig. 5). In fact, over this small depth range bioluminescence potential transitioned from contributing less than 3% of the pelagic photon budget to over 85%, and below 60 m bioluminescence contributed over 98% of the pelagic photon budget. The range of 20 m to 40 m also represents an ecotone for bioluminescent communities at this location in the polar night. Ecotones are transitional zones between patches of different and relatively homogenous ecological community types^42^, which often exhibit higher levels of biodiversity than surrounding patches^43^. Diversity (H′) of the bioluminescent community, albeit low at all depths was largest in the luminescent community at 40 m and deeper than in shallower depths (Fig. 4 and Table 3). Furthermore, the community at 40 m was more heterogeneously distributed than communities measured at other depths in the water column, requiring 30s longer to reach the half-saturation of maximum diversity during sampling (Fig. 4 and Table 3). Such an ecotone in Kongsfjord bioluminescent plankton during winter is consistent with other reports of zooplankton distribution in Svalbard fjords at this time of year, where their depth distribution is uneven with lower concentrations in the uppermost 25 m, increasing below 50 m^38^,^44^. The composition of this luminescent community, and its relationship to bioluminescence potential, is likewise similar to other studies from this and other regions of the Arctic throughout the year^16^,^23^,^29^,^32^. Specifically, lower bioluminescence potential was observed in shallow communities that were dominated by dinoflagellates, while higher bioluminescence potential occurred in deeper communities dominated by the copepod *M. longa*. One major difference is the depth distribution of dinoflagellates over seasons, which can account for up to 96% of bioluminescence in the upper 100 m of Norwegian fjords in summer^32^, but contributed 16–33% over these depths in our winter sampling. Our winter observations of the bioluminescent community suggest the influence of strong seasonal cycles in atmospheric irradiance on phytoplankton and zooplankton community dynamics in turn structure an ecotone in the bioluminescent community that coincides with a bioluminescence compensation depth where photons derived from internal bioluminescence sources begin to exceed those from atmospheric sources.

Here we demonstrate that the 20 m–40 m depth range in Kongsfjord is of particular interest for biological activity during the Arctic winter, a finding which has emerged in other polar night studies. Within this region of the water column zooplankton perform diel vertical migration^45^,^46^, and key zooplankton reach their visual threshold for the perception of scalar irradiance^33^. Our data now show an ecotone in the bioluminescent community at these depths as it transitions from a dinoflagellate dominated community to one dominated by copepods. We suggest this ecological structure is linked to a transition in the pelagic light field–from one dominated by dim, downwelling atmospheric irradiance to one dominated by bioluminescent point sources. Such transition zones, or bioluminescence compensation depths, can be defined as the depth range where bioluminescence overtakes atmosphere-derived light in contribution to the pelagic photon budget (Fig. 5). We posit these depths may be of particular importance to understanding how bioluminescence structures planktonic communities both in polar regions and at lower latitudes.

In the context of the Arctic winter, as one moves northwards the polar night increases in duration and the sun increases in angle below the horizon^34^, meaning that atmosphere-derived light is dimmer for an extended period of time and shallower depths will experience lower irradiance than they do in Kongsfjord. In these environments, bioluminescence will increase in relative contribution to the pelagic photon budget and the bioluminescence compensation depth will occur even shallower than they do in Kongsfjord. Moving further south from Kongsfjord, the light regime transitions from one that experiences a seasonal dark period to a diel light cycle with daytime irradiance that is brighter than in Kongsfjord during the winter^14^. In the Sargasso Sea, bioluminescence varies over the diel cycle and is greater during the night than during the day^15^. In summer, bioluminescence potential in the upper 150 m of the Sargasso Sea^47^ is on the order of 10^11 ^photons m^−3^ with 800 to 1500 bioluminescent emissions m^−3^, compared to our measurements of 10^13 ^photons m^−3^ and 693 emissions m^−3^ in Kongsfjord during winter. In ecosystems such as the Sargasso Sea, not only will bioluminescence compensation depths occur deeper, but they are also likely to move throughout the water column on a diel cycle as the sun rises and sets, zooplankton perform diel vertical migrations, and circadian cycles in luminescence are entrained. This vertically moving transition zone is likely a dynamic area of biological activity. Targeting it in future studies can provide a better understanding of how light structures the planktonic community and affects visually mediated behaviors (e.g., Fig. S1, Tables S1 and S2). However, it may prove difficult to differentiate taxa using *in situ* bathyphotometer approaches in locations with higher species diversity and abundance. But adaptive sampling with *in situ* bioluminescence profiling used to guide subsequent net or optical sampling could be used.

This study takes a first step in accounting for the depth-dependent dynamics of the bioluminescent community structure during the high Arctic winter. As in the deep sea, bioluminescence potential in the Arctic winter could play a large role in predator-prey dynamics, which we explore in a brief visual modeling example presented in the Supplementary Information (Fig. S1, Tables S1 and S2). We conclude that the transition zone between vertical habitat dominated by atmosphere-derived light to those dominated by bioluminescent light, defined as bioluminescence compensation depth, is an important habitat feature for further study.

## Methods

### Water column profiles

We conducted all sampling in Kongsfjord, Svalbard (78.936°N, 11.943°E) during January 2014. With an instrumented cage, we profiled from the surface to 120 m (bottom depth ≈ 200 m) twice at midday and twice at midnight over a 4-day period (n = 4 profiles). Instruments included a bathyphotometer (UBAT–Underwater Bioluminescence Assessment Tool, WetLabs, Philomath, OR) and CTD (SBE 49 FastCAT, Sea-Bird, Bellevue, WA). For each cast, we held instruments for 4 min at every 20 m depth interval to measure bioluminescence; prior work has shown such stops are necessary to characterize the bioluminescent community^14^,^16^. We determined the 4 min stop length used here from an analysis of the variation in bioluminescence potential (i.e. radiant energy produced by organisms in the UBAT in response to the turbulent stimulus) during a preliminary UBAT deployment at static depth.

### Identifying Bioluminescent Taxa with a Flash Kinetics Library

Ultimately, we determine the composition and vertical distribution of the winter bioluminescent community in Kongsfjord by isolating individual bioluminescent flashes from water column profiles and identifying the taxon that produced it according to flash kinetics. This analysis required development of a library of taxon-specific bioluminescent flash kinetics. We constructed this library in the laboratory by loading previously-identified plankton into a UBAT. Organisms were collected by net from three Svalbard fjords (Kongsfjord, Rijpfjord, and Billefjord) and marginal sea ice north of Svalbard (80.37°N, 11.31°E). We inserted plankton into the inflow of the UBAT one individual at a time for zooplankton and in groups of multiple individuals for phytoplankton in order to measure the kinetics of their emissions. Taxa tested for luminescence were: amphipods (*Themisto abyssorum, Themisto libellula*), appendicularians (*Oikopleura* spp., *Fritillaria borealis*), chaetognaths (*Parasagitta elegans*), cnidarians (*Aglantha digitale*), ctenophores (*Beroe cucumis, Mertensia ovum*), copepods (*Calanus* spp., *Metridia longa, Oithona* spp., *Paraeuchaeta* spp., *Triconia borealis* (=*Oncaea borealis*), dinoflagellates (*Protoperidinium* spp.), krill (*Meganyctiphanes norvegica, Thysanoessa inermis*), and ostracods (*Boroecia* spp.). Krill are counter-illuminators and required additional (gentle squeeze) before addition to the UBAT in order to generate flashes. We also re-analyzed existing UBAT data^29^, which increased the number of flashes for *Beroe cucumis, Metridia longa,* and *Meganyctiphanes norvegica.* Flash kinetic signatures drawn from existing data^29^ and from individuals tested in the current study did not differ (t-test, *P* > 0.05 for each species), so we treated all flashes for a given taxon as replicates.

For those taxa which consistently produced bioluminescence in the UBAT, flash kinetics were analyzed to develop a 4-parameter signature. These parameters were: the maximum bioluminescence produced at any point during the emission (BL_max_, photons s^−1^), the cumulative sum of bioluminescence until the maximum (∑_max_, photons s^−1^), the time until the emission reached maximum (T_max_, s), and the average bioluminescence produced during the emission (BL_mean_, photons s^−1^) (Fig. 1). Parameters were calculated for each replicate flash, and then averaged for each taxon (Table 1).

Collectively, the parameters calculated above represent a flash kinetic signature for each taxon which can be used to identify it based on emissions of bioluminescent zooplankton recorded *in situ* with the UBAT^29^. To identify the taxa responsible for producing *in situ* emissions measured during profiling, we determined the 4-parameter signature for each emission recorded during the first four minutes at profile stops, and calculated the error between emission signatures and each taxon-specific signature in the library. To do this, for each *in situ* emission captured by the UBAT during field deployment we first calculated the parameter errors (*φ*) with equation (1). These are defined as separate errors for each of the 4 parameters of the *in situ* emission with respect to parameters for each taxon signature in the library (BL_max_, BL_mean_, T_max_, and Σ_max_):


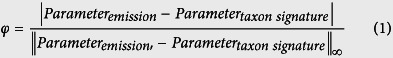


We then summed the parameter errors for each taxon and divided by the number of parameters to calculate the cumulative error (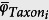) for each *in situ* emission against each taxon signature in the library with equation (2):


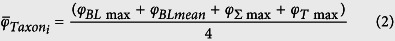


Finally, we compared 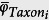 to the mean error for a given taxon determined from an analysis of the laboratory emissions used to create the bioluminescence library (Φ_*Taxon*_) with equation (3):


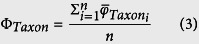


If 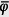 for an *in situ* emission was below Φ_*Taxon*_+ 1SD for a given taxon in the bioluminescence library, it was identified as that taxon. We compared *in situ* emissions to taxonomic signatures in the bioluminescence library iteratively in the following order: *Beroe cucumis, Boroecia* spp., *Metridia longa, Mertensia ovum, Thysanoessa* spp., *Meganyctiphanes norvegica,* and dinoflagellates. We chose this iterative method over identifying an emission as the taxon for which it had the smallest error^29^ because the iterative approach produced the highest number of correct identifications during preliminary analysis of a dataset for which species were known. With our approach, some emissions could not be classified as any taxon in the library, and were considered unidentified. Only *in situ* emissions which had a BL_max_ greater than 3 × 10^8 ^photons (100*x* that of background seawater) and which were not compound were identified. Compound emissions were defined as those with multiple peaks and a minimum between peaks that was either below BL_mean_, or 5-fold less than BL_max_. They were excluded from analysis because a true BL_max_ could not be determined.

### Analysis of bioluminescent community composition

We calculated taxonomic abundances from the number of individuals identified as each of the 7 taxa in the bioluminescence library and the volume of water pumped by the UBAT during each stop (89.5 L). From these data, we used a series of Two-way ANOVAs with time-of-day and depth as factors to test for the effect of local time at sampling (midday vs. midnight) on: (1) the abundance of individual taxa (Two-way ANOVA, *P* > 0.05), (2) the total number of individuals sampled (Two-way ANOVA, F_14,27_ = 0.786, *P* = 0.6), and (3) the total bioluminescence potential (Two-way ANOVA, F_14,27_ = 1.639, *P* = 0.2). Accordingly, the four casts were considered replicates for future analyses. To quantify differences in community structure at depth intervals, we calculated Shannon diversity^48^ and Pielou’s evenness^49^ indices for each 4 min sample. For each replicate, we also calculated Shannon diversity at 30 s intervals during the 4-min sampling period and fit each depth interval with a nonlinear regression model based on a Michaelis-Menten function. The maximum diversity, H′_max_, and the time to half-maximum diversity, K, were calculated from the regression for each depth in order to understand the diversity and heterogeneity of each depth-stratified community.

### Atmospheric irradiance in Kongsfjord

Diffuse skylight was measured adjacent to Kongsfjord on 25 January 2015 for input into a radiative transfer model using Hydrolight 5.2 RTE^50^, as described in Cohen *et al*.^33^. Diffuse spectral irradiance was measured at midday in Ny-Ålesund on 25 January 2015 using a QE Pro spectrometer (Ocean Optics, FL, USA) that received 180° of diffuse skylight reflected from a Spectralon reflectance reference plate (Labsphere Inc., NH, USA). This was used as input to model the underwater light field throughout the water column for clear conditions with Raman scattering and Chlorophyll-*a* fluorescence of 0.06 μg L^−1^ over the whole water column. Inherent optical properties in Kongsfjord necessary for radiative transfer modeling were measured in January 2015 using a Wet Labs ac-9 absorption/scattering meter^33^. Downwelling irradiance from 395 nm to 695 nm was modeled in 5nm increments for every meter to 99 m.

## Additional Information

**How to cite this article**: Cronin, H. A. *et al*. Bioluminescence as an ecological factor during high Arctic polar night. *Sci. Rep.* **6**, 36374; doi: 10.1038/srep36374 (2016).

**Publisher’s note:** Springer Nature remains neutral with regard to jurisdictional claims in published maps and institutional affiliations.

## Supplementary Material

Supplementary Information

## Figures and Tables

**Table 1:** Library of flash kinetic parameter signatures (±SE) for taxa collected around Svalbard during 2012 and 2014.

Taxon	Location	BL_max_	BL_mean_	∑_max_	T_max_
Dinoflagellates					
* Protoperidinium* spp. (n = 7)	Rijpfjord, Billefjord	1.14 ± 0.27	0.37 ± 0.079	2.86 ± 0.73	0.08 ± 0.003
Ctenophores					
* Beroe cucumis* (n = 16)	Kongsfjord	200.05 ± 48.43	65.45 ± 18.16	3176.5 ± 1557	0.52 ± 0.078
* Mertensia ovum* (n = 13)	Rijpfjord	2.05 ± 0.41	0.66 ± 0.13	17.21 ± 4.51	0.34 ± 0.066
Copepods					
* Metridia* spp. (n = 13)	Kongsfjord	6.47 ± 1.29	1.70 ± 0.32	19.69 ± 3.47	0.13 ± 0.037
Krill					
* Meganyctiphanes norvegica* (n = 6)	Kongsfjord	12.98 ± 6.90	3.59 ± 1.80	85.98 ± 38.84	0.15 ± 0.046
* Thysanoessa inermis* (n = 18)	Kongsfjord	25.32 ± 7.63	12.61 ± 3.63	209.97 ± 69.39	0.22 ± 0.022
Ostracods					
* Boroecia* spp. (n = 3)	Sea ice drift	76.65 ± 39.11	22.24 ± 11.10	323.27 ± 146.82	0.34 ± 0.19

Sample size is in parentheses with each taxon. BL_max_, BL_mean_, and ∑_max_ are reported × 10^9 ^photons s^−1^, and T_max_ is reported in seconds.

**Table 2:** Mean abundances (individuals m^−3^, ± SE) of each taxon in the bioluminescence library for UBAT profiles (n = 4) (see Fig. 3).

Depth (m)	Dinoflagellates	Ctenophores		Copepods	Krill		Ostracods
	*Protoperidinium* spp.	*Mertensia ovum*	*Beroe cucumis*	*Metridia* spp.	*Meganyctiphanes norvegica*	*Thysanoessa* spp.	*Boroecia* spp.
1	119.56 ± 6.98	72.08 ± 18.62	0	71.63 ± 10.86	2.79 ± 2.79	0	5.59 ± 5.59
20	122.88 ± 27.36	92.16 ± 25.54	0	114.50 ± 28.98	2.79 ± 2.79	5.59 ± 3.22	2.79 ± 2.79
40	114.50 ± 11.51	108.91 ± 18.45	0	164.77 ± 12.38	16.76 ± 9.67	30.72 ± 5.35	0
60	108.91 ± 16.04	106.12 ± 16.12	0	226.21 ± 15.38	22.34 ± 11.17	19.55 ± 9.54	5.59 ± 3.22
80	122.88 ± 13.68	103.33 ± 12.38	2.79 ± 2.79	231.79 ± 37.15	25.13 ± 2.79	22.34 ± 10.18	5.59 ± 3.22
100	100.54 ± 12.07	58.65 ± 9.54	5.59 ± 3.22	242.96 ± 17.88	22.34 ± 7.99	30.72 ± 5.35	8.38 ± 5.35
120	114.50 ± 21.08	75.40 ± 12.38	2.79 ± 2.79	192.69 ± 31.10	22.34 ± 11.17	27.93 ± 11.17	8.38 ± 5.35

Abundances are calculated from the first 4 minutes (89.5 L) sampled.

**Table 3:** Shannon diversity (H′) and Pielou’s evenness (J′) for the entire 4 min UBAT sampling period at each depth (mean, n = 4), and maximum Shannon diversity (H′_max_) and the time (s) to reach ½ of the maximum diversity (K) calculated from non-linear regression of Shannon diversity at 30s intervals during UBAT profiles.

Depth (m)	H′	J′	H′_max_ (95% CI)	K (95% CI)
1	1.11	0.57	1.19(1.0–1.3)	14.1(0.16–28.1)
20	1.14	0.59	1.09(1.0–1.2)	11.6(5.2–18.1)
40	1.33	0.69	1.69(1.5–1.9)	57.7(33.4–82.0)
60	1.29	0.66	1.47(1.4–1.6)	27.2(15.6–38.7)
80	1.35	0.69	1.42(1.3–1.5)	14.8(7.9–21.8)
100	1.34	0.69	1.50(1.3–1.7)	23.0(4.2–41.8)
120	1.25	0.64	1.42(1.3–1.5)	12.0(5.3–18.6)
