# Supplementary material for: Bioluminescence as an ecological factor during high Arctic polar night
Source: Sci Rep. 2016 Nov 2;6:36374. doi: 10.1038/srep36374 (PMC5090458; doi:10.1038/srep36374)
Supplement: Supplementary Information [file srep36374-s1.pdf]

Supplementary Information for:

Bioluminescence as an ecological factor during high Arctic polar night

Heather A. Cronin[1], Jonathan H. Cohen[1*], Jørgen Berge[2,4], Geir Johnsen[3,4], Mark A. Moline[1]

[1] University of Delaware, School of Marine Science and Policy, Lewes, DE 19958 United Sates of America

[2] UiT The Arctic University of Norway, Faculty of Biosciences, Fisheries and Economics, Dept of Arctic and Marine Biology, N-9037 Tromsø, Norway

[3] Norwegian University of Science and Technology, Dept. biology, Trondheim Biological Station, N-7491, Trondheim, Norway

[4] University Centre in Svalbard, Pb 156, N-9171 Longyearbyen, Norway

*Corresponding author: Jonathan H. Cohen, jhcohen@udel.edu, 1-302-645-4298

Example: How polar night bioluminescence distributions could influence ecological interactions

Here we present one example of how *in situ* data on bioluminescence produced by the planktonic community might be used to explore pelagic trophic interactions. We modeled the effect of bioluminescence on visual perception of an ecologically significant predator-prey pair. Specifically, we considered the maximum range at which the krill *Thysanoessa inermis* could detect a predatory little auk. *Thysanoessa inermis* is both a dominant micronekton species in Kongsfjord during winter and other times of year[1-5], and is a member of the bioluminescent community measured in the current study. Little auks are resident year-round on Svalbard and are active predators on *T. inermis* in Kongsfjord during winter, often feeding selectively on krill[3,5]. Mechanical stimulation of bioluminescence by diving little auks as they move through the water column would present a visual stimulus for krill. Accordingly, we modeled krill visual range for perception of a little auk diving through the bioluminescent community that we observed in Kongsfjord.

We used equations from Nilsson et al.[6] parameterized by our own observations of the environmental light field (downwelling radiance derived from HydroLight modelling and bioluminescent light derived from UBAT measurements) and krill visual perception (derived from both electrophysiological recording from *T. inermis* eyes and histology, e.g., Cohen et al.[7]) to quantify visual range for the krill superposition compound eye detecting the silhouette of a little auk approaching from above and therefore appearing as an extended black target triggering bioluminescence against downwelling space-light. Visual range for a krill at a given depth viewing an oncoming diving little auk can be solved for with equation (1):

(1) $$\left|N_{bio} + N_{black} + N_{space}\right| = R\sqrt{N_{bio} + N_{black} + N_{space} + 2X_{ch}}$$

where N_{bio} is the mean photon count originating from bioluminescent sources, N_{black} is the mean photon count from light scattered into the line of sight between the visual target (little auk) and the observer (krill), N_{space} is the mean photon count from background space-light, *R* is a reliability coefficient for photon capture, and X_{ch} is the number of false photons per integration time (i.e. photoreceptor noise in the observer's eye). The component equations for each of the terms in our equation (1) are provided here in Supplementary Table 1. Input variables for these calculations are listed in Supplementary Table 2. While patchy distributions of luminescent organisms may occur in nature, we do not have sufficient data to parameterize that here and have assumed a homogeneous distribution of luminescent organisms for each 20 m depth bin measured by the UBAT. Further, as our approach excludes ~25-30% of emissions at each depth as compound or unidentified (e.g. Fig. 3b), our parameterization of bioluminescence is likely an underestimation of the bioluminescent community. Opposing that, however, we assume an equal capacity for mechanically stimulating luminescence between the UBAT's high turbulence and the impact of a diving little auk. It is likely that birds are less efficient at evoking luminescence than the UBAT making our quantification of bioluminescence an overestimate in this respect. Collectively, there is uncertainty in the value of bird-stimulated bioluminescence, but our parameterization of it here provides a useful starting point.

We calculated visual range for krill positioned at 1 m depth increments from 1 to 99 m depth using three different scenarios for the stimulated bioluminescent community in order to test whether variations in bioluminescent community composition altered visual performance. These included scenarios of: (1) no bioluminescence throughout the entire water column; and (2) the average depth-stratified Kongsfjord luminescent community as measured by UBAT at 20 m intervals. In both visual models, the distance at which krill could not discriminate between the

little auk and the background was set at twice the wingspan of a little auk, 0.76m, or the point at which it subtended more than 28° of the visual field[6].

The modelling scenario of the visual interactions between krill and little auks supports that bioluminescence emitted from the planktonic community observed in Kongsfjord during the polar night is sufficient to influence predator-prey interactions in the epipelagic. In the upper 20m of the water column, downwelling atmospheric light is amply bright to cause a diving little auk to appear as a dark silhouette against the ambient background when viewed from below by a krill. The bird's dark silhouette would be apparent to its krill prey irrespective of any bioluminescence induced by the moving bird. But luminescence dominated by dinoflagellates, in this case stimulated by the body/wings of the moving bird, is analogous to counterillumination[8] in that it weakens the contrast of the bird's silhouette against downwelling atmospheric light. At slightly deeper depths coincident with the bioluminescence compensation depth, when atmospheric light has been attenuated and bioluminescence is the dominant source of photons (25-30m in the present study), the optical situation changes. The visual range for krill perceiving a diving bird rapidly increases at depths below 30m, where both the luminescent community composition changes from dinoflagellates to copepods, and the quantity of bioluminescent emissions increase. At these depths, krill view little auks or by extension other predators, in reverse contrast as bioluminescent emissions illuminate their body[9]. Net and acoustic studies of krill vertical distribution in Kongsfjord during polar night[1] suggest krill populations are predominantly at depths between 30-60m. Numerous factors beyond the scope of this brief example could explain the observed vertical distribution (e.g., krill visual threshold[7], non-luminous prey abundance[10], etc.), but at a minimum our modeling example suggests residence below the bioluminescence compensation depth represents a visually beneficial habitat for krill.

Visual detection of a predator does not guarantee a krill's ability to behaviorally escape predation, and this may be particularly true when krill reside at shallower depths. There, even though predators may theoretically be visible against downwelling spacelight, the ability of krill to behaviorally respond and escape predation may be limited in optical conditions dominated by atmospheric light where predator foraging behavior is enhanced[11], and in turn krill avoid them.

A major limitation of our analysis is that the visual model employed here is based on visual discrimination of the target against the background during one integration time, and therefore is best applied to longer viewing distances such that collections of bioluminescent point sources appear as a steady glow. The spatial resolution of the superposition eye in *T. inermis* is fairly poor (interommatidial angle [$\Delta\phi$] = 3.8°), but its critical flicker fusion frequency of 20 Hz suggests that temporally it may be capable of perceiving individual light flashes. Taken together, it is not clear whether bioluminescence disrupted from a moving bird would be perceived by krill as a steady glow or as individual flashes over the relatively short visual ranges (<10m) modeled here. Additionally, the background light field was only modeled for one time of day – midday – which is the brightest background light. A more complete consideration of this example requires further attention to spatial and temporal capabilities of krill, variation in ambient and bioluminescent light, and also the visual function of birds viewing krill targets.

References

1. Berge, J., Båtnes, A.S., Johnsen, G., Blackwell, S.M., & Moline, M.A. Bioluminescence in the high Arctic during the polar night. *Mar. Biol.* **159**, 231-237 (2012).

2. Berge, J. *et al.* In the dark: paradigms of Arctic ecosystems during polar night challenged by new understanding. *Prog. Oceanogr.* **139**, 258-271 (2015a).

3. Berge, J. *et al.* Unexpected levels of biological activity during the polar night offers new perspectives on a warming Arctic. *Curr. Biol.* **25**, 2555-2561 (2015b).

4. Buchholz, F., Buchholz, C. & Weslawski, J.M. Ten years after: krill as indicator of changes in the macro-zooplankton communities of two Arctic fjords. *Polar Biol.* **33**, 101-113 (2010).

5. Huenerlage, K., Graeve, M., Buchholz, C. & Buchholz, F. The other krill: overwintering physiology of adult *Thysanoessa inermis* (Euphausiacea) from the high-Arctic Kongsfjord. *Aquat. Biol.* **23**, 225-235 (2015).

6. Nilsson, D.E., Warrant, E. & Johnsen, S. Computational visual ecology in the pelagic realm. *Philos. Trans. R. Soc. Lond. B Biol. Sci.* **369**, 20130038 (2014).

7. Cohen, J.H. *et al.* Is ambient light during the high Arctic polar night sufficient to act as a visual cue for zooplankton? *Plos One* **10**, e0126247 (2015).

8. Harper, R.D. & Case J.F. Disruptive counterillumination and its anti-predatory value in the plainfish midshipman *Porichthys notatus*. *Mar. Biol.* **134**, 529-540 (1999).

9. Nilsson, D.E., Warrant, E.J., Johnsen, S., Hanlon, R. & Shashar, N. A unique advantage for giant eyes in giant squid. *Curr. Biol.* **22**, 683-688 (2012).

10. Hays, G.C. A review of the adaptive significance and ecosystem consequences of zooplankton diel vertical migrations. *Hydrobiologia.* **503**, 163-170 (2003).

11. Lovvorn, J.R. Modeling profitability for the smallest marine endotherms: auklets foraging within pelagic prey patches. *Aquat. Biol.* **8**, 203-219 (2010).

12. Clark, P.J. & Evans, F.C. Generalization of a nearest neighbor measure of dispersion for use in k-dimensions. *Ecology* **60**, 316-317 (1979).

13. Behrens, K., Cox, C. *Seawatching: Eastern waterbirds in flight*. (Houghton Mifflin Harcourt, 2013).

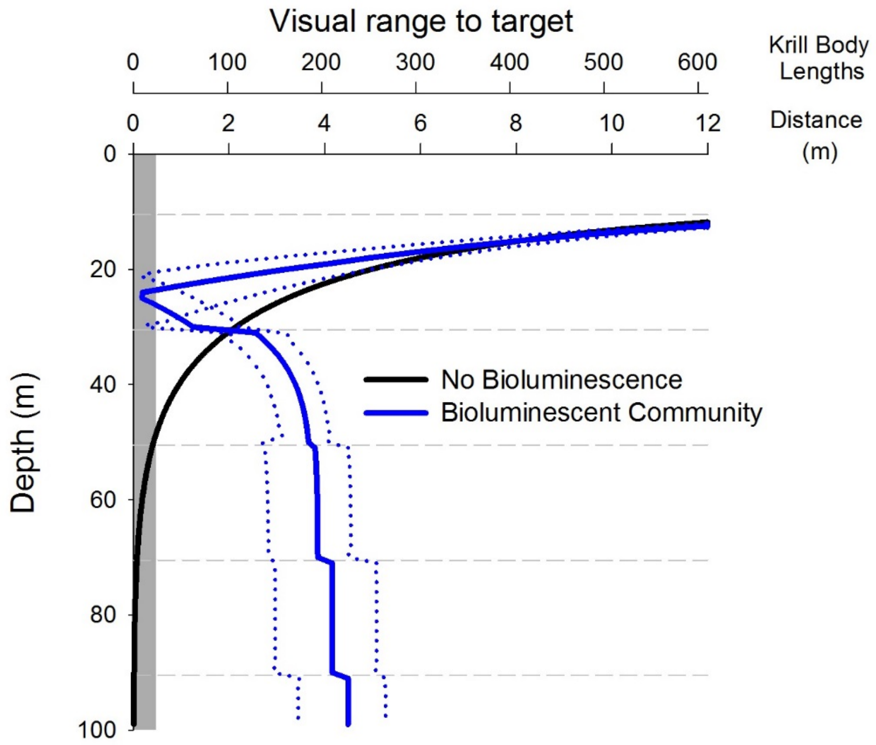

Supplementary Figure 1. Visual range of a krill (*Thysanoessa inermis*) viewing a little auk (*Alle alle*) approaching from above. A scenario with no bioluminescence in the water column between the krill and bird (black line) is compared to one in which bioluminescence is present (blue lines). The solid blue line shows the model with a mean bioluminescent community composed of the taxa observed in our Kongsfjord UBAT profiles, with each taxon emitting light at intensities recorded by that instrument. Dotted blue lines are similar models run with luminescent communities defined by the upper and lower bounds for abundance of each luminescent taxa. Gray horizontal dashed lines represent depths at which the input bioluminescent community for the model was adjusted to reflect depth-specific changes community in Kongsfjord. The shaded box (visual ranges ≤0.76m) represents krill visual ranges where the little auk subtended greater than 28° of the krill's visual field. At these visual ranges, krill photoreceptors cannot simultaneously view both the little auk and the background, and therefore discrimination of the little auk is not possible.

Supplementary Table 1: A list of component equations for visual models of the detection of an extended black target triggering bioluminescence and a description of their purposes. Equations are from Nilsson et al.[6], except $N_{bio,taxon}$ which is from Clark and Evans[12]. Variable definitions and their values are listed in Supplementary Table 2.

Description	Equation
N_{space}; the mean photon count originating from background space light	$0.617A^2(\frac{T}{r})^2 q\Delta t * I_{space}$
N_{black}; the mean photon count originating from light scattered into the line of sight	$0.617A^2(\frac{T}{r})^2 q\Delta t * I_{space}(1 - e^{(\kappa-\alpha)r})$
N_{bio}; the mean photon count originating from all bioluminescent sources	$\Sigma N_{bio_{taxon}}$
$N_{bio\ taxon}$; the mean photon count originating from a single bioluminescent taxon	$P_{taxon}\left(\frac{E_{taxon}A^2}{16r^2}\right)e^{-\alpha * r}q\Delta t$
x; the average distance between bioluminescent point sources across an extended object	$\frac{0.55397}{\rho^{1/3}}$
Xch; the number of false photons per integration time	$(\frac{Tf}{rd})^2 X\Delta t$
P_{taxon}; total number of point sources from each taxon in the bioluminescent community viewed by the target pixel	$\frac{\pi T^3}{2.86x^3}$

Supplementary Table 2: Variables used in visual models and their values.

Variable	Value	Units	Source
**R**: reliability coefficient	1.96		set for a 95% confidence interval[6]
**T**: width of target	0.381	m	Wingspan of a little auk[13]
**f**: focal length	0.000145	m	Measured from sagittal sections of *T. inermis* eyes
**r**: range to target	--	m	Found for every output depth using GRG nonlinear optimization of equation (1)
**d**: photoreceptor diameter	0.0000096	m	Measured from sagittal sections of *T. inermis* eyes
**X**: dark noise per photoreceptor	0.000028	photons s^{-1}	[6]
**Δt**: integration time	0.05	s	Based on CFF_{max} of *T. inermis* eye at 1 °C = 20 Hz (J.H. Cohen, unpubl. data)
**A**: pupil diameter	0.000549	m	Measured from ½ of *T. inermis* eye diameter
**q**: detection efficiency	0.36		[6]
$\mathbf{I_{space}}$: radiance of background space light in the direction of view	--	photons m^{-2} s^{-1} sr^{-1}	Modeled in Hydrolight (5.2 RTE), weighted by the spectral sensitivity of *T. inermis*[3]
**κ**: attenuation coefficient of background radiance	0.166	m^{-1}	Modeled at 495nm using Hydrolight (5.2 RTE)
**α**: beam attenuation coefficient of seawater	0.147	m^{-1}	Average “c” value at 488nm from an ac-9 profile in Kongsfjord in January 2015[7]
**ρ**: taxon abundance	--	Individuals m^{-3}	Measured in UBAT profiles for each taxon and variable by taxon and depth bin
